# The complete chloroplast genome of *Cynometra cebuensis* F. Seid. (Fabaceae), a critically endangered endemic plant from Cebu, Philippines

**DOI:** 10.1080/23802359.2025.2544681

**Published:** 2025-08-11

**Authors:** Jeremaiah L. Estrada, Claudette I. Canonigo, Kristine Joyce O. Quiñones, Renerio P. Gentallan

**Affiliations:** ^a^ Department of Biology and Environmental Science, College of Science, University of the Philippines Cebu, Cebu City, Philippines; ^b^ Crop Breeding and Genetic Resources Division, Institute of Crop Science, College of Agriculture and Food Science, University of the Philippines Los Baños, Laguna, Philippines; ^c^ Department of Science and Technology, Science Education Institute, Taguig, Philippines

**Keywords:** Conservation, Detarioideae, endemism, phylogeny, taxonomy

## Abstract

Cynometra cebuensis F. Seid., a critically endangered species endemic to Cebu, Philippines, lacks published genomic data. We present its first complete chloroplast genome, assembled using Illumina paired-end sequencing. The 158,474 bp genome includes a large single-copy (86,821 bp), small single-copy (19,329 bp), and two inverted repeats (26,162 bp each), with a GC content of 36.4%. It contains 125 genes: 80 protein-coding, 37 tRNA, and eight rRNA. Phylogenetic analysis places C. cebuensis in a monophyletic group within Detarioideae, closely related to C. lenticellata, providing essential data for its identification, phylogeny, and conservation.

## Introduction

*Cynometra cebuensis* F. Seid is an endemic and critically endangered tree species within the family Fabaceae (DENR-DAO 2017-11; Department of Environment and Natural Resources 2017; IUCN 2025), first documented in the Tabunan Forest of Cebu City, Philippines (Seidenschwarz 2013). Commonly known as ‘nipot-nipot,’ *C. cebuensis* is characterized by its distinctive juvenile inflorescences – single-branched, bright pink tassels – that mature into a creamy, greenish-yellow hue. This species is slow-growing and predominantly occurs in limestone forest ecosystems, typically between 400 and 600 m above sea level (Seidenschwarz 2013; Estrada et al. 2024). This species is classified in the subfamily Detarioideae, as defined by de la Estrella et al. (2018), with the updated generic circumscription provided by Radosavljevic et al. (2017). This classification follows the new legume subfamily framework proposed by the Legume Phylogeny Working Group, which recognizes six subfamilies within Leguminosae (LPWG 2017).

Taxonomic classification of *C. cebuensis* remains challenging, primarily due to the absence of easily identifiable flowers and the predominance of juvenile individuals within its natural habitat. Moreover, the flowering period of mature trees is brief, lasting only a few days, further complicating identification efforts (Estrada et al. 2024). Despite its critical conservation status, *C. cebuensis* has been relatively understudied, with limited available data regarding its geographical distribution, potential uses, and pharmacological properties. Published literature primarily focuses on its morphological traits and restricted distribution in Cebu, with significant contributions from Seidenschwarz (2013), Lillo et al. (2023), and Pelser et al. (2011). However, comprehensive research on this species remains sparse, highlighting the need for further investigation into its ecology and potential applications.

Although several closely related species with a cosmopolitan distribution have been sequenced (Bruneau et al. 2001; de la Estrella et al. 2017; Radosavljevic et al. 2017; Zhang et al. 2020), the chloroplast genome of *C. cebuensis* remains unsequenced. Detailed genetic studies on this species are still limited, likely due to the restricted size of its wild populations, which is a consequence of its critically endangered status. As such, the present study aims to characterize and sequence the complete chloroplast genome of *C. cebuensis*. This genomic information is crucial for understanding its evolutionary and phylogenetic relationships within the Fabaceae family, providing a foundation for informed conservation strategies and enhancing efforts to protect this rare and endangered species.

## Materials and methods

### Entry protocol, ethical considerations, and compliance with IUCN conservation guidelines

Prior to the collection of *C. cebuensi*s, Wildlife Gratuitous Permits (No. 2024-09 and 2024-22) were secured from the Department of Environment and Natural Resources Region 7 in compliance with the Philippine Republic Act No. 9147 (Wildlife Resources Conservation and Protection Act) (FAO 2025). All sampling was conducted in strict adherence to the guidelines established by the International Union for Conservation of Nature (IUCN), the Convention on Biological Diversity, and the Convention on International Trade in Endangered Species of Wild Fauna and Flora (CITES) (IUCN 1989) to ensure that no harm would be caused to existing populations. Three branches of the *C. cebuensis* were collected from Mt. Lantoy in Argao, Cebu (9° 54′ 8.9994″ N and 123° 31′ 46.9992″ E) and from the Experimental Forest Station of the DENR-Ecosystems Research and Development Bureau-Coastal Resources and Ecotourism Research, Development and Extension Center (DENR-ERDB-CRERDEC) in Minglanilla, Cebu (10° 19′ 31.0008″ N and 123° 46′ 27.9984″ E) (Figure S1).

### Collection and taxonomic identification

During the fieldwork, the species was primarily identified with the help of forest guards and local guides. The identity of the species was further verified using published papers by Seidenschwarz (2013) and Lillo et al. (2023). Co’s Digital Flora of the Philippines (Pelser et al. 2011) was also used to confirm the species presence in the areas. Branches of *C. cebuensis* were collected to serve as voucher specimens (ICROPS1519), which were deposited in the Philippine Herbarium of Cultivated Plants of the Institute of Crop Science at UPLB (https://cafs.uplb.edu.ph/icrops/, Curator Dr. Renerio P. Gentallan Jr., rpgentallan@up.edu.ph).

### DNA isolation, chloroplast genome sequencing, assembly, and annotation

Silica-dried leaves of *Cynometra cebuensis* were used for DNA extraction via a modified CTAB method (Quiñones, Gentallan, Bartolome, Madayag, Timog, et al. 2023). DNA concentration and quality were assessed using a DeNovix DS-11+ spectrophotometer (A260/280) and 1% agarose gel electrophoresis, respectively. Sequencing was performed by NovogeneAIT Genomics Singapore using the Illumina HiSeq-PE150 platform (San Diego, CA). After quality filtering with Fastp v0.20.0 (Chen et al. 2018), 25,165,616 clean 150-bp paired-end reads were obtained and assembled into a circular chloroplast genome using GetOrganelle v1.7.5+ (Jin et al. 2020). Coverage was visualized with Bowtie2 in Geneious Prime. Annotation was done using CPGAVAS2 (Shi et al. 2019), and genome maps showing genes, repeats, and splicing genes were generated using CPGView (Liu et al. 2023). The complete genome is available in GenBank (PQ790067.1), BioProject (PRJNA1126608), and BioSample (SAMN41947244).

### Genome comparison and phylogenetic analysis

To compare and characterize the contraction and expansion of inverted repeats (IRs) at the junction site among *Cynometra* chloroplast genomes, IRScope (Amiryousefi et al. 2018) was implemented. The complete chloroplast sequences of *C. cebuensis*, three other *Cynometra* species, and representatives from the new subfamily classification of the Leguminosae family were downloaded from NCBI. The sequences were aligned using MAFFT (Katoh and Standley 2013). A maximum-likelihood (ML) tree was constructed with the General Time Reversible, Gamma (GTR + G) model (Nei and Kumar 2000) using MEGA-X (Kumar et al. 2001), which included 1000 bootstrap replicates.

## Results

### Morphological characteristics of the plant material

**Figure 1.**
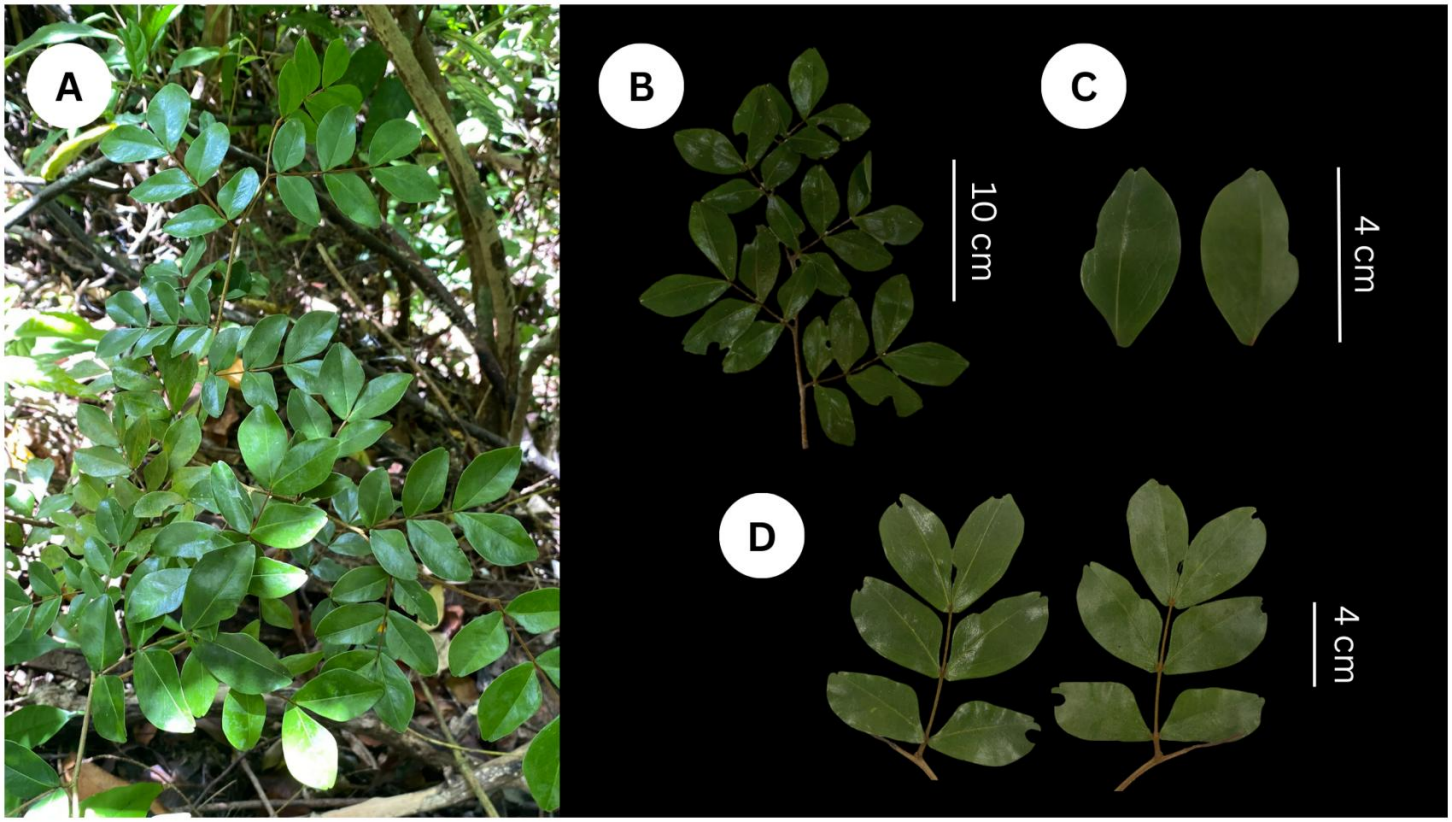
*C. cebuensis* wildling (A) in the dry forests of Cebu, Philippines, with its leaflets (B) showing the adaxial and abaxial sides (C, D). The voucher specimens (ICROPS 1519) were deposited in the Philippine Herbarium of Cultivated Plants, Institute of Crop Science, University of the Philippines Los Baños (https://cafs.uplb.edu.ph/icrops/, Curator Dr. Renerio P. Gentallan Jr., rpgentallan@up.edu.ph). All photos in this study were original and taken *in situ* by Jeremaiah L. Estrada.

*Cynometra cebuensis* F. Seid is a tropical forest tree that grows up to 12 m in height and is characterized by its distinctive leaf morphology (Figure 1(A)). Its leaves consist of (3–)4–6 leaflets attached to petioles that are rugose, hairy, and with an approximate length of 5–7 mm. Its leaflets are dark green, sessile, arranged in pairs, and range from 2 to 5(–6) by 0.8–1.5(–2.2) cm in size. Moreover, these have ovate to oblong in shape, emarginate apex, and an asymmetric base that is cuneate on the acroscopic side and rounded to auriculate on the basiscopic side (Figure 1(B–D)). Its margin is thickened and its midrib is 5–7 mm from the acroscopic margin with about 7–9 pairs of lateral veins (Lillo et al. 2023; Estrada et al. 2024). In terms of habitat, *C. cebuensis* is present in dry forests over limestone terrain at elevations of 400 m above sea level or higher. The observed characteristics of the collected reference plant material were similar to the studies of Seidenschwarz (2013) and Lillo et al. (2023).

### Chloroplast genome characteristics of *C. cebuensis*

**Figure 2.**
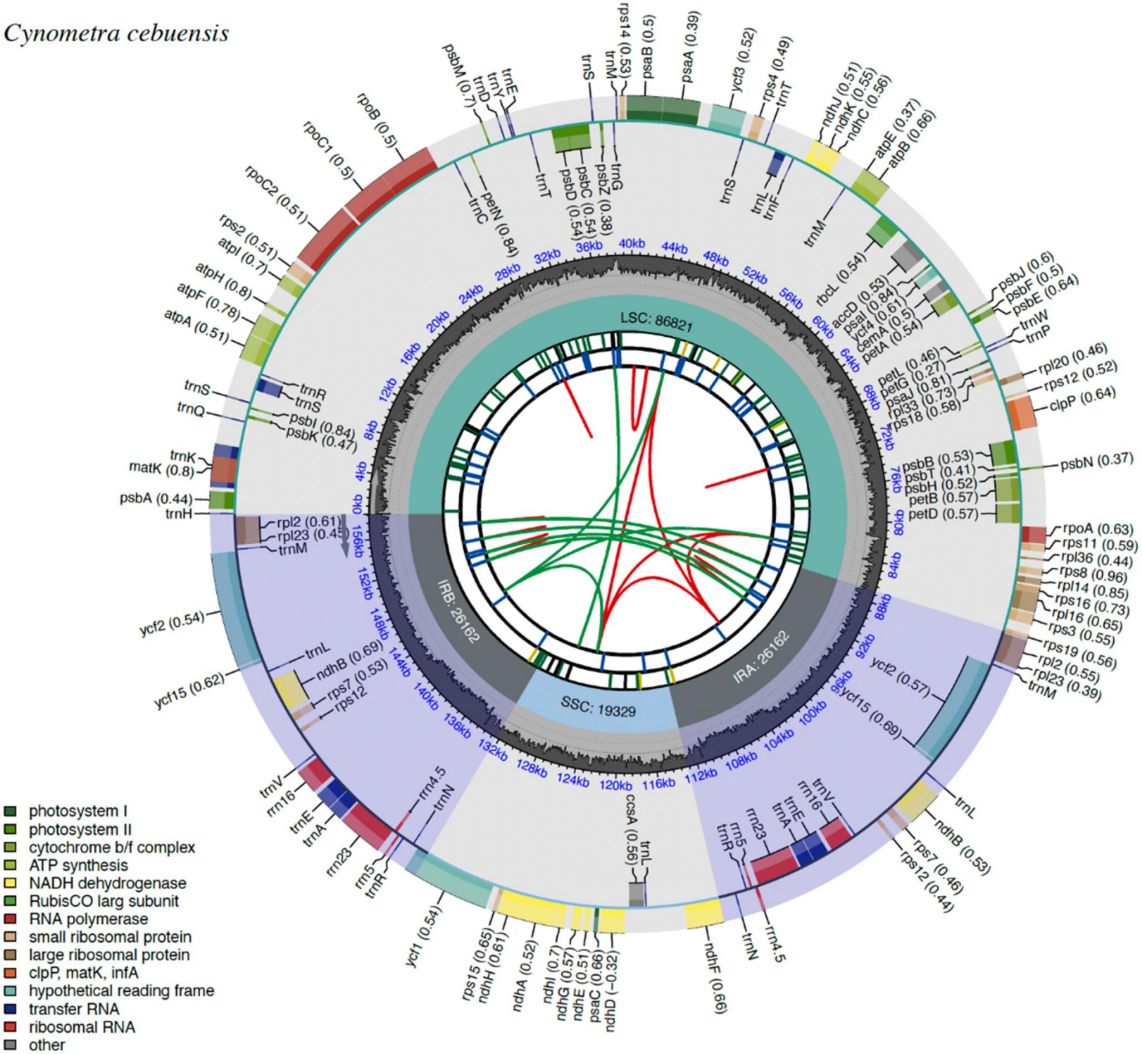
This schematic map, generated using CPGView, shows the overall features of the complete chloroplast genome of *C. cebuensis*. The innermost circle depicts dispersed repeats, which include forward repeats (connected by red arcs) and palindromic repeats (connected by green arcs). The second circle illustrates long tandem repeats represented by short blue bars. In the third circle, simple sequence repeats are represented by short colored bars, each indicating a different repeat unit size (RUS): black for complex repeats (c), green for RUS = 1 (p1), yellow for RUS = 2 (p2), purple for RUS = 3 (p3), blue for RUS = 4 (p4), orange for RUS = 5 (p5), and red for RUS = 6 (p6). The fourth circle delineates regions including small single-copy (SSC), inverted repeats (IRa and IRb), and large single-copy (LSC) regions. The fifth track plots the GC content across the plastome. In the sixth circle, plastome genes are annotated, with optional codon usage bias noted in parentheses after each gene name. Genes are color-coded by their functional classification as indicated in the legend. Genes in the inner and outer circles are transcribed clockwise and counterclockwise, respectively.

The circularized complete chloroplast genome sequence of *C. cebuensis* spans 158,474 bp, adhering to the typical quadripartite structure of plastomes: a pair of IR regions (IRa and IRb, each 26,162 bp), a large single-copy region (LSC, 86,821 bp), and a small single-copy region (SSC, 19,329 bp) (Figure 2) with an average depth of coverage of ×1017 (Figure S2).

*C. cebuensis* had 80 protein-coding sequences (CDS), 37 tRNA genes, eight rRNA genes, and 125 total genes (Table S1). The schematic map of *rps*12 (Figure S3), a trans-splicing gene, depicts three distinct exons, two of which are duplicated because they are located in the IR regions. Also, a schematic map of 14 cis-splicing genes (*atpF*, *rpoC1*, *ycf3*, *clpP*, *petB*, petD, *rpl16*, *rpl2*, *ycf2*, *ndhB*, *ndhA*, *ndhB*, *ycf2*, and *rpl2*) in the chloroplast genome was visualized (Figure S4). The assembled chloroplast genome of *Cynometra cebuensis* serves as a molecular reference for species identification, particularly in the absence of reproductive characters. This can help identify emerging populations, particularly wildlings (Estrada et al. 2024) and regenerants due to timber harvesting during dry season of *C. cebuensis* (Seidenschwarz 2013), which we hypothesize to be overlooked due to absence of their reproductive parts.

The chloroplast genome of *C. cebuensis* measured 158,474 base pairs, slightly shorter than that of *C. ramiflora* (159,812 bp; NC_047332.1; Zhang et al. 2020). In contrast to *C. ramiflora*, the chloroplast genome of *C. cebuensis* had a longer IR region but shorter LSC and SSC regions. Nevertheless, both species exhibited identical ACGT base counts and frequencies of tRNA (37) and rRNA (8) genes.

### Phylogenetic analysis

**Figure 3.**
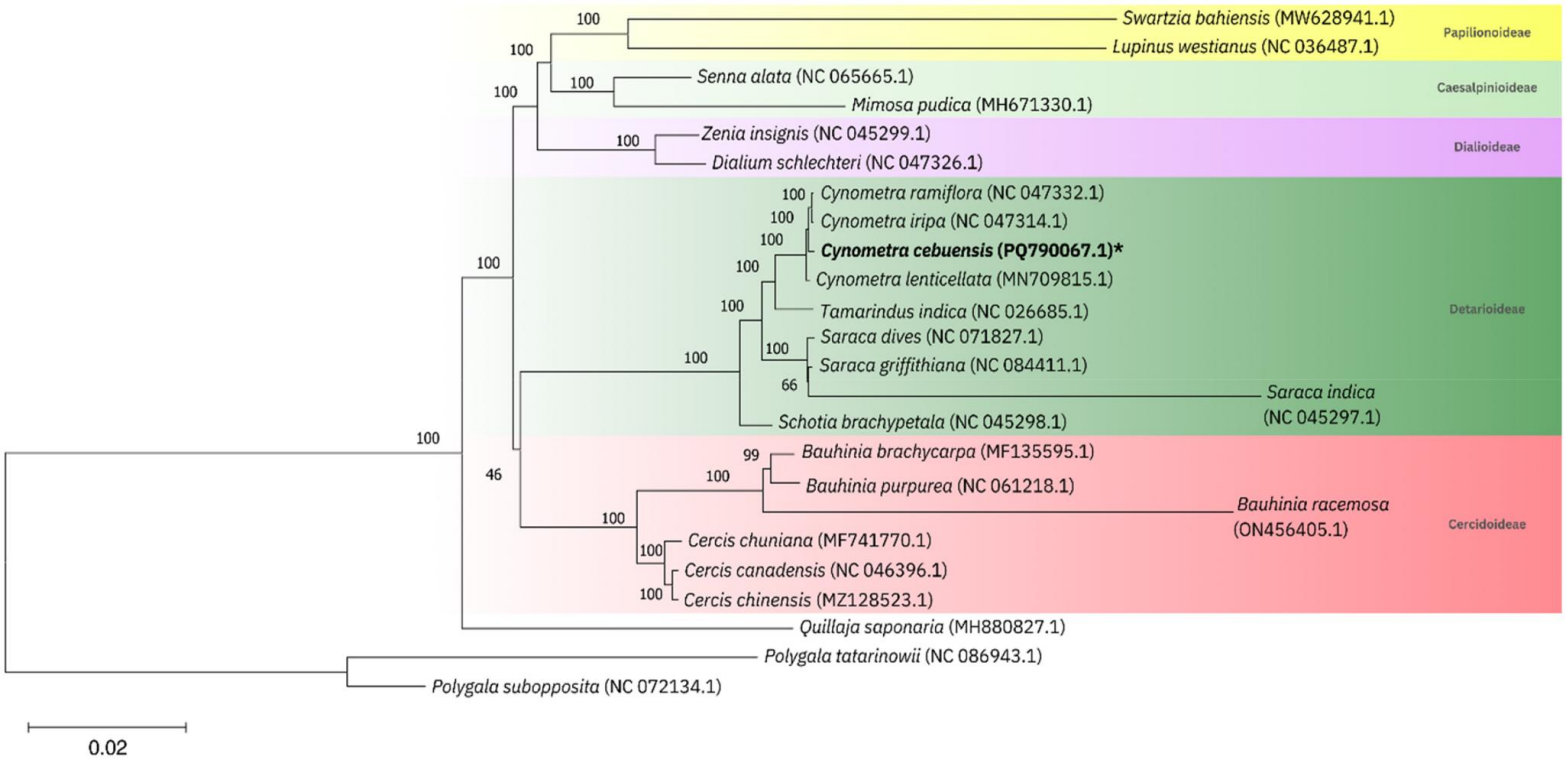
Maximum-likelihood (ML) tree generated in MEGA X using the complete chloroplast genomes of the following Fabaceae species which were available in the NCBI GenBank: *Swartzia bahiensis* (MW628941.1; Lee et al. 2021), *Lupinus westianus* (NC_036487.1, Xu et al. 2017), *Senna alata* (NC_065665.1; Quiñones, Gentallan, Bartolome, Madayag, Vera Cruz, et al. 2023), *Mimosa pudica* (MH671330.1; Yang et al. 2018), *Zenia insignis* (NC_045299.1; Koenen et al. 2020), *Dialium schlechteri* (NC_047326.1; Zhang et al. 2020), *Cynometra ramiflora* (NC_047332.1; Zhang et al. 2020), *Cynometra cebuensis* (PQ790067.1, this study), *Cynometra iripa* (NC_047314.1; Zhang et al. 2020), *Cynometra lenticellata* (MN709815.1; Zhang et al. 2020), *Tamarindus indica* (NC_026685.1; Sabir et al. 2017), *Saraca dives* (NC_071827.1; Liao 2023), *Saraca griffithiana* (NC_084411.1; Jiayu 2023), *Saraca indica* (NC_045297.1; Koenen et al. 2020), *Schotia brachypetala* (NC_045298.1; Koenen et al. 2020), *Bauhinia brachycarpa* (MF135595.1; Wang et al. 2018), *Bauhinia purpurea* (NC_061218.1; Zhang 2022), *Bauhinia racemosa* (ON456405.1; Xiao et al. 2022), *Cercis chuniana* (MF741770.1; Liu et al. 2018), *Cercis canadensis* (NC_046396.1; Feng et al. 2019), *Cercis chinensis* (MZ128523.1; Hou 2021), *Quillaja saponaria* (MH880827.1; Vizoso et al. 2018), and the outgroup from the Polygalaceae family: *Polygala tatarinowii* (NC_086943.1; Wang 2024), *Polygala subopposita* (NC_072134.1; Wang 2023).

The generated phylogenetic tree exhibited a topology consistent with the phylogram produced from the analysis of peptide sequences from 81 plastid-encoded proteins (LPWG 2017) (Figure 3). The subfamily Detarioideae formed a close evolutionary relationship with the subfamily Cercidoideae. Within the Detarioideae subfamily, *Cynometra* spp. and *Tamarindus indicus* shared the most recent common ancestor, as they belong to the same tribe, Amherstieae, whereas *Schotia brachypetala* exhibited a more distant relationship due to its placement in the tribe Schotieae. The observed relationship between the different taxonomic groups in this subfamily is concordant with the topology of the new phylogeny-based tribal classification of the subfamily Detarioideae, derived from the analysis of three combined plastid loci (de la Estrella et al. 2018).

Furthermore, the genus *Cynometra* formed a monophyletic group, supported by a high bootstrap value of 100%. Within this monophyletic group, *C. ramiflora* and *C. iripa* shared the most recent common ancestor, while *C. cebuensis* was their sister taxon. The node support value of 100% further corroborates the robust inference of evolutionary relationships among the selected species. This tree demonstrates that *C. cebuensis* is distinct from both the native species of *Cynometra* and other related species, supporting the potential of the established reference chloroplast genome as a molecular super-barcode for species identification of this critically endangered island-endemic species.

## Discussion and conclusions

This study presents the first complete chloroplast genome of *Cynometra cebuensis* F. Seid., a critically endangered tree species endemic to the island of Cebu, Philippines. The plastome length of 158,474 bp and its quadripartite structure – composed of a LSC, a SSC, and two IRs – is consistent with the general organization of angiosperm chloroplast genomes (Wicke et al. 2011). The GC content of 36.4% also falls within the typical range for Fabaceae chloroplast genomes, suggesting structural and compositional conservation within the family (Jansen and Ruhlman 2012).

The presence of 125 unique genes, including 80 protein-coding genes, 37 tRNA genes, and eight rRNA genes, reflects a gene content comparable to that of other legumes in subfamily Detarioideae (LPWG 2017; Radosavljevic et al. 2017). Notably, the structure and gene composition of the *C. cebuensis* plastome reinforce its phylogenetic placement and provide a genetic baseline for future population-level studies.

Phylogenetic reconstruction placed *C. cebuensis* within a monophyletic clade of the subfamily Detarioideae, closely aligned with *Cynometra lenticellata*. This result supports earlier morphological classifications (Bruneau et al. 2001; de la Estrella et al. 2017) and highlights the potential of plastid genomes in resolving taxonomic uncertainties in Detarioideae and other legume lineages. The relatively close relationship with *C. lenticellata*, an Australian species, may suggest historical biogeographic connections or retained ancestral traits within the genus *Cynometra*, warranting further investigation using nuclear and mitochondrial genomes.

From a conservation perspective, the sequencing of the plastome of *C. cebuensis* is a timely and crucial step. Given its extremely limited distribution and fragmented habitat, the availability of genetic information can inform conservation management through precise species identification, assessments of genetic diversity, and development of molecular markers (Hollingsworth et al. 2011). Moreover, plastome data may facilitate the identification of cryptic lineages or potential hybrids, particularly important in disturbed forest remnants where closely related taxa may co-occur.

In conclusion, this study not only fills a critical gap in genomic resources for *C. cebuensis* but also reinforces the value of plastid genomes in understanding evolutionary relationships and supporting conservation of endangered taxa. Future work should integrate population genomics and ecological assessments to develop a holistic strategy for the long-term survival of *C. cebuensis* in its native habitat.

## Supplementary Material

Supplemental Material

## Data Availability

The genome sequence data that support the findings of this study are openly available in GenBank of the National Center for Biotechnology Information (NCBI) at https://www.ncbi.nlm.nih.gov/nuccore/2889538505 with accession number PQ790067.1. The associated BioProject, SRA, and BioSample numbers are the following: BioProject (PRJNA1126608), SRA (SRP576884), and BioSample (SAMN41947244), respectively.
