# Supplementary material for: The complete chloroplast genome of Cynometra cebuensis F. Seid. (Fabaceae), a critically endangered endemic plant from Cebu, Philippines
Source: Mitochondrial DNA B Resour. 2025 Aug 11;10(9):809–15. doi: 10.1080/23802359.2025.2544681 (PMC12340937; doi:10.1080/23802359.2025.2544681)
Supplement: Supplemental Material [file TMDN_A_2544681_SM5454.docx]

Supplementary Materials

**Figure S1.** Collection points of *Cynometra cebuensis* within Mt. Lantoy in Argao, Cebu, and the Experimental Forest Station of DENR-CRERDEC in Minglanilla, Cebu.
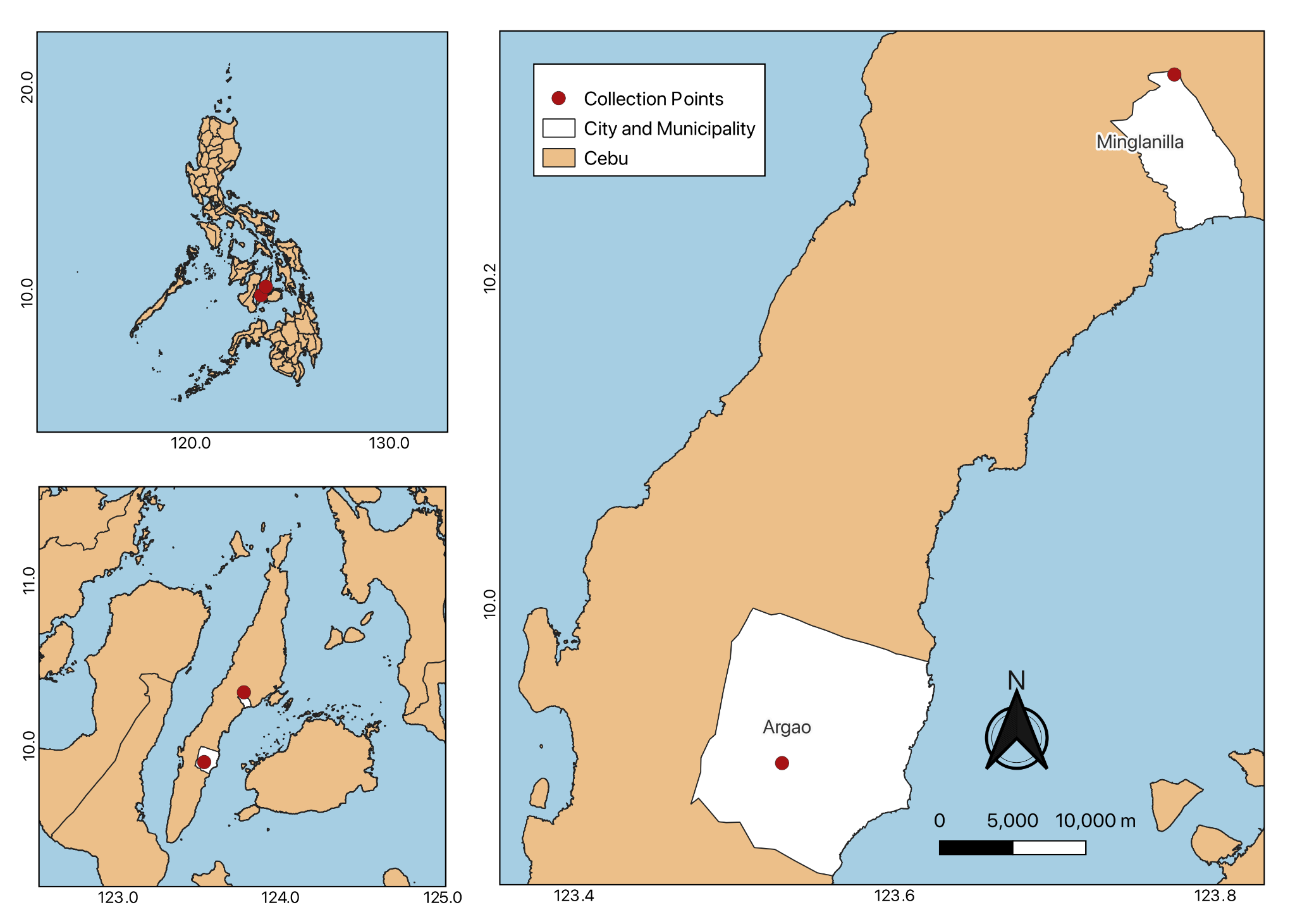


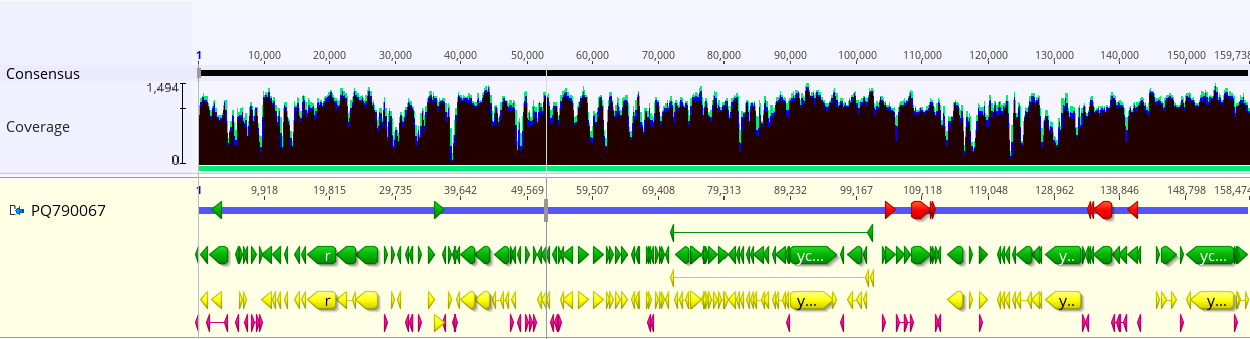


A

B

**Figure S2.** Depth of coverage plot (A) and physical map of cpDNA (B). The reads from Illumina NGS sequencing were remapped on the cp genome sequence of *Cynometra cebuensis* (PQ790067) using Bowtie2 as implemented in Geneious Prime 2024 with minimum sequencing depth of 47, average sequencing depth of 1017, and maximum sequencing depth of 1494.

**
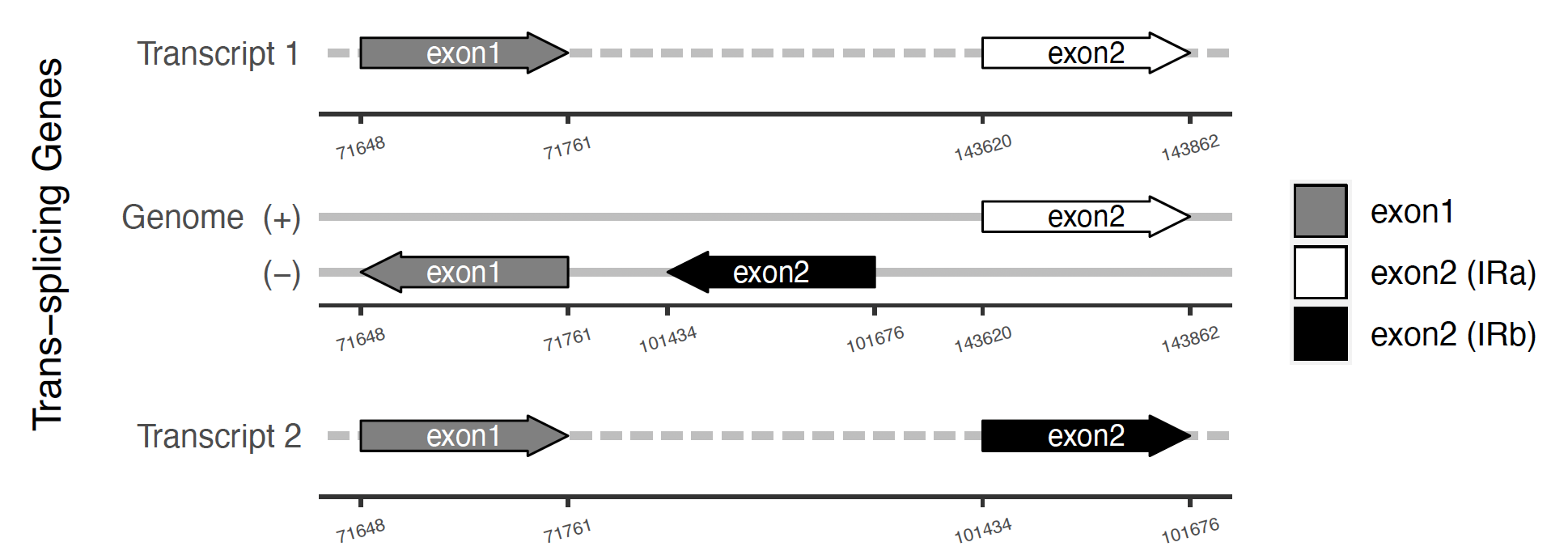
**

**Figure S3.** Schematic map of the trans-splicing gene, *rps12*, in the *C. cebuensis* plastome having three unique exons with two of them being duplicates within the IR regions using its GenBank file through CPGView.


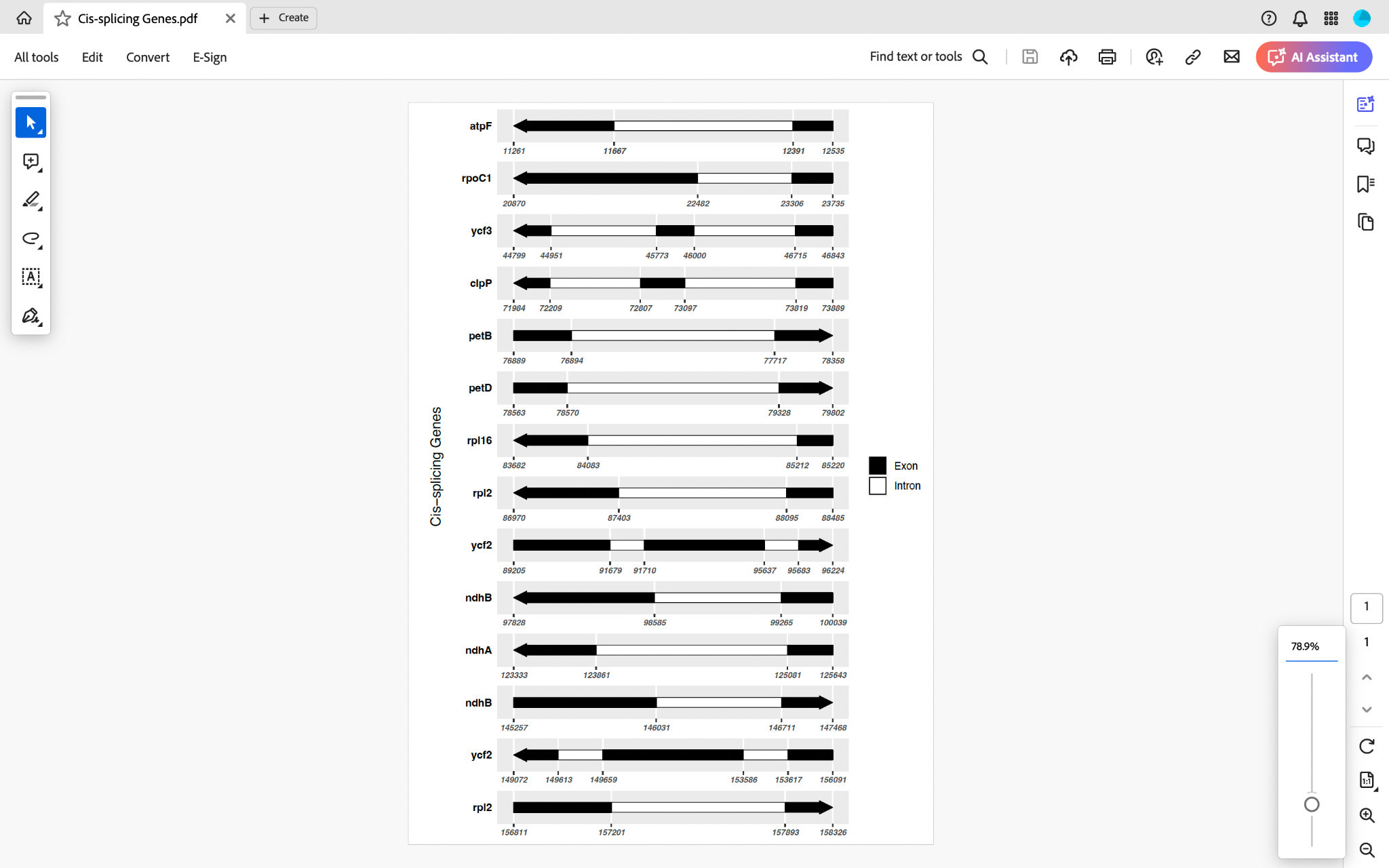


**Figure S4.** Schematic map of the cis-splicing genes in the *C. cebuensis* genome, arranged from top to bottom respective to their order in the genome. The gene names are written on the left of their respective structures on the right. The exons and introns are black and white, respectively. The arrow indicates the sense direction of each gene. The lengths of exons and introns are not drawn to scale.

**Table S1.** The *de novo*-assembled *Cynometra cebuensis* chloroplast genome showed varying genomic features when compared with other published plastomes of *Cynometra* distributed in the Philippines.

| **PLASTOME FEATURE** | ***C. cebuensis***  (this study) | ***C. ramiflora**** | ***C. iripa**** |
| --- | --- | --- | --- |
| GenBank No. | PQ790067 | NC_047332.1 | NC_047314.1 |
| Size (bp) | 158,474 | 159,812 | 159,685 |
| LSC (bp) | 86,821 | 88,153 | 88, 026 |
| SSC (bp) | 19,329 | 19, 413 | 19, 413 |
| IR (bp) | 26,162 | 26,123 | 26,123 |
| A (%) | 31.3 | 31.3 | 31.3 |
| C (%) | 18.5 | 18.5 | 18.5 |
| G (%) | 17.9 | 17.9 | 17.9 |
| T (%) | 32.3 | 32.3 | 32.3 |
| Total genes | 125 | 128 | 128 |
| CDS | 80 | 83 | 83 |
| tRNA | 37 | 37 | 37 |
| rRNA | 8 | 8 | 8 |

*Zhang et al. (2020)
